# Impact of correlated inputs to neurons: modeling observations from *in vivo* intracellular recordings

**DOI:** 10.1007/s10827-014-0502-z

**Published:** 2014-05-03

**Authors:** Man Yi Yim, Arvind Kumar, Ad Aertsen, Stefan Rotter

**Affiliations:** 1 Department of Mathematics, University of Hong Kong, Pokfulam Road, Hong Kong; 2 Bernstein Center Freiburg, Faculty of Biology, University of Freiburg, Hansastr. 9a, 79104 Freiburg, Germany

**Keywords:** Input correlation, Rate modulation, Correlation transfer, Temporal structure, Barrel cortex

## Abstract

*In vivo* recordings in rat somatosensory cortex suggest that excitatory and inhibitory inputs are often correlated during spontaneous and sensory-evoked activity. Using a computational approach, we study how the interplay of input correlations and timing observed in experiments controls the spiking probability of single neurons. Several correlation-based mechanisms are identified, which can effectively switch a neuron on and off. In addition, we investigate the transfer of input correlation to output correlation in pairs of neurons, at the spike train and the membrane potential levels, by considering spike-driving and non-spike-driving inputs separately. In particular, we propose a plausible explanation for the *in vivo* finding that membrane potentials in neighboring neurons are correlated, but the spike-triggered averages of membrane potentials preceding a spike are not: Neighboring neurons possibly receive an ongoing bombardment of correlated subthreshold background inputs, and occasionally uncorrelated spike-driving inputs.

## Introduction

Input correlations have been observed in membrane potentials recorded from neurons in various brain regions. At the single neuron level, the existence of “activity bumps” (that is, large fluctuations) in the membrane potential trace indicates the arrival of coordinated inputs within a narrow time window (Okun and Lampl 2008; DeWeese and Zador 2006). In dual *in vivo* intracellular recordings in rodent barrel cortex, membrane potentials of pairs of simultaneously recorded neurons were reported to be correlated in both quiet and whisking states (Poulet and Petersen 2008; Gentet et al. 2010). Furthermore, instantaneous correlations of excitatory and inhibitory inputs have been observed in the membrane potential of neurons in the rodent barrel cortex (Okun and Lampl 2008) and the retina (Cafaro and Rieke 2010) in *in vivo* recordings.

Here, we studied the impact of the temporal structure of synaptic inputs, both on the response firing rate and on the response spike correlation of neurons—two quantities that are widely investigated in neuronal dynamics. Previous analytical work has treated various aspects of input correlations such as higher order statistics of the input (Bernander et al. 1994; Kuhn et al. 2003; Schultze-Kraft et al. 2013), the neuronal integration of excitatory and inhibitory inputs (Kuhn et al. 2004), and the time constant of input currents (Svirskis and Rinzel 2000; Moreno et al. 2002) on output firing rates. Gating of signal representations by correlated inputs has been illustrated in network simulations (Kremkow et al. 2010). Modulation of the activity level of a neuron by background synaptic noise statistics has been demonstrated in *in vitro* experiments (Sceniak and Sabo 2010).

Here, we studied the interplay of multiple potential rate modulating factors observed in experiments, and identified several scenarios which lead to a strong effect on firing rate. In addition, the input-triggered coordination of the outputs of pairs of neurons is less well explored, and some more complex phenomena in this context are not understood at all. For example, it turns out that correlations at the level of spikes and subthreshold membrane potentials can both be independently regulated by inputs. Intriguingly, membrane potentials of neighboring neurons were found to be highly correlated (Okun and Lampl 2008; Poulet and Petersen 2008; Gentet et al. 2010), but their spike responses were not (Gentet et al. 2010). Earlier theoretical work (de la Rocha et al. 2007; Shea-Brown et al. 2008; Tchumatchenko et al. 2010; Rosenbaum and Josic 2011) considered a pair of neurons receiving correlated inputs, leading to membrane potential correlations. Several important insights into the mechanisms of output spike correlation and their underlying membrane potential correlations (Kriener et al. 2008; Krumin and Shoham 2009) are offered by this model, some of which were verified experimentally (de la Rocha et al. 2007). By contrast, in spike-triggered averaged membrane potentials of excitatory neuron pairs in a layer 2/3 barrel column, depolarization in a neuron was found to be exceedingly small when another, nearby neuron was depolarized strongly towards firing an action potential (cf. Fig 4e in Poulet and Petersen 2008 and Fig 6G, left, in Gentet et al. 2010). We will argue that this small depolarization is much smaller than predicted by the commonly used Poisson model.

We reconstructed the situation in a more general model comprising two single neurons, based on observations made in *in vivo* intracellular recordings from somatosensory cortex (Okun and Lampl 2008). In this simple input-output scenario, we identified several factors based on the correlation structure and relative timing of the input spike trains that can either increase or decrease neuronal firing rates and correlations at all levels. Specifically, we suggest an extended perspective to reconcile the above-mentioned conflicting observations of correlated subthreshold membrane potential and uncorrelated spiking activity, by distinguishing between spike-driving (SD) and non-spike-driving (NSD) inputs. In this scheme, when two neurons receive independent SD inputs, the depolarization in one neuron can be small, whereas the other depolarizes strongly and fires an action potential, as observed in experiments (Poulet and Petersen 2008; Gentet et al. 2010). Furthermore, SD and NSD inputs can induce output spike and membrane potential correlations that are independent of each other. Thus, our results provide a more complete understanding of how the temporal structure of inputs shapes both the response firing rate and the spike correlation of neurons.

## Methods

### Neuron model

The neuron model used in this work is the leaky integrate-and-fire (LIF) neuron with conductance-based synapses. Its subthreshold dynamics of the membrane potential *V*(*t*) is described by the equation

1 $$ C\, \frac{d}{dt}V(t) + G_{L} \, \left[V(t) - V_{r}\right] = I(t) $$

where *I* is the total synaptic input current to the neuron and *C*, *G*_*L*_ and *V*_*r*_ reflect the passive cell properties: capacitance, leak conductance and resting membrane potential, respectively. When the membrane potential reaches a fixed spiking threshold *V*_th_, a spike is emitted. Then the membrane potential is reset to its resting value and a brief pause *t*_ref_ for synaptic integration is imposed to mimic the refractory period in real neurons.

Both excitatory and inhibitory synaptic inputs are modeled by transient conductance changes using conductance transients that are described by an alpha function

2 $$ g_{E}(t) = \left\{ \begin{array}{ll} J_{E}\,\frac{t}{\tau_{E}}\,e^{1 - \frac{t}{\tau_{E}}} & \;\text{for}~t \geq 0\\ 0 & \;\text{for}~t<0, \end{array}\right. $$

and

3 $$ g_{I}(t) = \begin{array}{ll} J_{I}\,\frac{t}{\tau_{I}}\,e^{1 - \frac{t}{\tau_{I}}} & \;\text{for}~ t \geq 0\\ 0 & \;\text{for}~t<0, \end{array} $$

where *τ*_*E*_ and *τ*_*I*_ are the rise times for the excitatory and inhibitory synaptic inputs, respectively. The peak amplitudes *J*_*E*_ and *J*_*I*_ of the conductance transients are considered as the strength of the two types of synapses, respectively.

By assuming homogeneous couplings, the total excitatory conductance *G*_*E*_(*t*) in a neuron is given by

4 $$ G_{E}(t) = \sum_{k}g_{E}(t-t_{E,k}). $$

The sum runs over the sequence of spikes from excitatory sources (*t*_*E*,*k*_) impinging on the neuron. Similarly, the total inhibitory conductance is

5 $$ G_{I}(t) = \sum_{k}g_{I}(t-t_{I,k}). $$

The total synaptic current into a neuron is

6 $$ I(t) = - G_{E}(t)\,\left[V(t)-V_{E}\right] - G_{I}(t)\,\left[V(t)-V_{I}\right], $$

with *V*_*E*_ and *V*_*I*_ denoting the reversal potentials of the excitatory and inhibitory synaptic currents, respectively.

The parameter values were chosen to be consistent with the experimental literature (McCormick et al. 1985; Troyer and Miller 1997): *C*=500 pF, *G*_*L*_=25 nS, *V*_*r*_=−65 mV, *V*_th_=−50 mV and *t*_ref_=2 ms. We set *V*_*E*_=0 mV and *V*_*I*_ = −70 mV (Koch 1999). The rise times of the excitatory and inhibitory synapses were set to be 0.3 ms and 2 ms, and the peak conductances were *J*_*E*_=*J*_*I*_=15 nS, respectively. All simulations were carried out using a Python interface to NEST (Gewaltig and Diesmann 2007). The differential equations were integrated using the GNU Scientific Library (GSL) implementation of the 4th-order classical Runge-Kutta algorithm with adaptive step-size control. The output was generated at intervals of 0.1 ms. In the study on rate and correlation modulation, the simulation results were averaged over 5 trials of 20 seconds each.

### Input configurations

We considered different types of structured input to disentangle the different observations made in various *in vivo* experiments.

#### Correlated excitation and inhibition using MIP

**Fig. 1:**
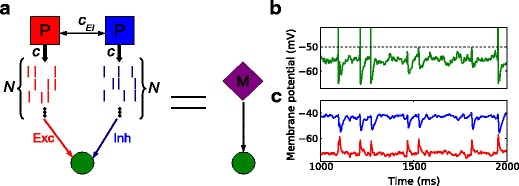
Scheme of the neuron model receiving correlated inputs. **a** A scheme of the correlation model. Two Poisson processes with correlation *c*_*EI*_ were used as the mother processes for excitatory and inhibitory inputs, respectively. Two ensembles of *N* Poisson spike trains, each with pairwise correlation *c*, generated by the MIP (multiple interacting process; see Kuhn et al. 2003) scenario, represent the source of input to a LIF neuron with conductance-based synapses. Red, blue and purple colors indicate excitation, inhibition and a mixture of both, respectively. **b** Sample membrane potential trace of the model neuron receiving correlated excitatory and inhibitory inputs. The dotted line indicates the spike threshold. The spike waveforms above threshold were manually inserted for aesthetic reasons. **c** Depolarizing and hyperpolarizing currents were injected into the neuron to isolate IPSPs (*blue, upper*) and EPSPs (*red, lower*), respectively. The response of the simulated neurons closely resemble the *in vivo* recordings in Okun and Lampl (2008)

was the input structure implemented as shown in Fig. 1a for the study of rate modulation of neurons in somatosensory cortex (Okun and Lampl 2008). First, we constructed two Poisson processes with correlation coefficient *c*_*EI*_, and then generated *N*=1000 spike trains by copying spikes from each of the mother processes with copy probability *c* as the excitatory and inhibitory inputs, respectively. This scheme is known as the multiple interaction process (MIP) (Bernander et al. 1994; Kuhn et al. 2003; Yim et al. 2011). Hence, *c*_*EI*_ characterizes the coupling strength between the excitatory and inhibitory inputs, while *c* is the correlation between excitatory or inhibitory spike trains that is responsible for the amplitude of the compound PSPs and, thereby, the distribution of membrane potential fluctuations. As a consequence, the correlation between individual excitatory and inhibitory spike trains amounts to *c* *c*_*EI*_.

#### Fluctuation-driven inputs

are a mixture of a Poisson excitatory synaptic input of rate *r*_*E*_ = 2000 Hz and a Poisson inhibitory synaptic input of rate *r*_*I*_ = 1647 Hz that are independent of each other. These values yield an output firing rate of around 1 Hz. If the degree of overlap between individual PSPs is high enough, as is the case here, the input statistics approaches that of a Gaussian white noise under the diffusion approximation (Rice 1944). In this scenario, spikes are mainly generated by membrane potential fluctuations (Kuhn et al. 2004). This scheme explains the irregular spiking throughout the cortex at all rates (Softky and Koch 1993; Shadlen and Newsome 1998; van Vreeswijk and Sompolinsky 1996).

#### Spike-driving (SD) inputs

are modeled as a set of *N*=1000 correlated excitatory spike trains using MIP with big enough *c* (in our case, *c*=0.05) projecting onto a neuron, such that most input spike clusters reliably evoke an output spike. A pair of correlated SD inputs are two correlated excitatory MIPs, generated in the same way as correlated excitation and inhibition using MIP as described above. Jittered input spikes were implemented by adding a uniformly distributed random number to the MIP inputs. The amount of jitter to individual spikes was chosen identical for common SD inputs to the neuron pair, in view of our model of two neighboring neurons sharing some common inputs. The support of the distribution is 30 ms, roughly fitting the observations made in the barrel cortex (Gentet et al. 2010). The SD input rate was tuned such that the output firing rate was around 1 Hz.

#### Non-spike-driving (NSD) inputs

are similar to the fluctuation-driven inputs as described above, but with a reduced excitatory drive *r*_*E*_=1400 Hz. Such an input provides a fluctuating background, but is in itself not sufficient to evoke a spike. We used this scenario in both rate and correlation modulation study.

In the following, we adopt the word “correlation” to describe the between-pool correlation, i.e. the correlation between different input pools projecting to individual neurons (Rosenbaum et al. 2010; Yim et al. 2011), unless specified otherwise. Among MIP inputs, correlation can be defined between the constituting spike trains, which we refer to as “within-pool correlation” (Rosenbaum et al. 2010; Yim et al. 2011).

### Data analysis

Throughout this work, we characterized the correlation of our measured quantities, which were either two membrane potential traces or two mildly low-pass filtered spike trains, using standard Pearson correlation coefficients. In addition, we tested our results for a range of parameters of the neuron model and its inputs, ascertaining ourselves that they were qualitatively robust to parameter changes.

#### Output spike correlation

Given two spike trains, we first lowpass filtered them with a triangular kernel of support (base length) 5 ms, and then computed the correlation coefficient.

#### Membrane potential correlation

Although there is no explicit spike waveform in the LIF model, the reset after the threshold crossing does induce a dramatic drop in the membrane potential. Inclusion of membrane potential reset will influence the estimate of the membrane potential correlation. Moreover, membrane potential correlation in real neurons is often computed after removing spikes, which is achieved either experimentally by hyperpolarizing the recorded neuron with a direct current to prevent it from spiking or, afterwards in the data analysis, by cutting out the spikes and their after-effects before the correlation analysis. Therefore, here, if one of the two neurons fired a spike, the membrane potential traces of both neurons were “cleaned” by removing 50 ms of the signal after the positive-going threshold crossing.

## Results

### Correlated MIP inputs can describe experimental observations

Figure 1b shows a sample membrane potential trace of the model neuron receiving correlated excitatory and inhibitory inputs as shown in Fig. 1a. Using this correlation model as the input source, we injected a depolarizing and a hyperpolarizing current into the model neuron, as described in *in vivo* intracellular recordings in the barrel cortex of lightly anesthetized rats (Okun and Lampl 2008; Atallah and Scanziani 2009). IPSPs and EPSPs were revealed by applying depolarizing and hyperpolarizing currents, respectively, as illustrated in Fig. 1c. This simulation result closely resembles the experimental observations.

### Rate modulation by temporal structure of inputs

**Fig. 2:**
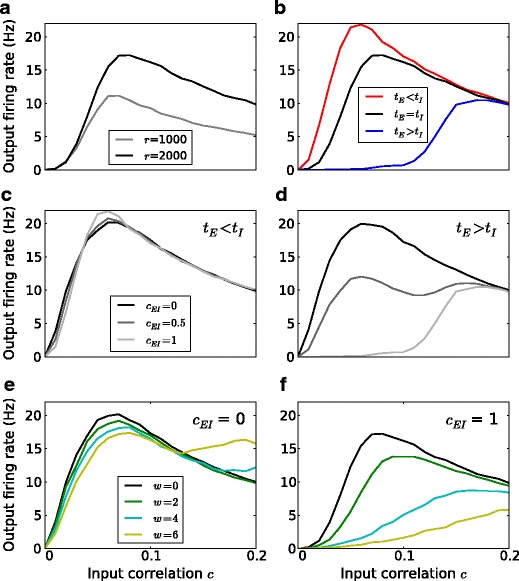
Rate modulation by temporal structure of input spike trains. Mean output firing rate as a function of input correlation: **a** for different input rates *r*, **b** for different relative timing between excitatory *τ*_*ex*_ and inhibitory inputs *τ*_*in*_; here we used a relative time lag of 2 ms if present and *c*_*EI*_ = 1, **c** for different coupling *c*_*EI*_ between excitation and inhibition with inhibition preceding excitation, **d** for different coupling *c*_*EI*_ between excitation and inhibition with excitation preceding inhibition, **e** for different amount of input jitter *w* with coupled excitation and inhibition, **f** for different amount of input jitter *w* with uncoupled excitation and inhibition

Based on our interpretations of the *in vivo* intracellular recordings from somatosensory cortex (Okun and Lampl 2008), we identified the interplay of several factors which might be employed in the neocortex or other brain regions to modulate output firing rates. In view of the fact that the amplitudes of compound PSPs can vary and the simultaneously measured EPSP and IPSP sizes exhibit a linear relation (Okun and Lampl 2008), we assume that excitatory and inhibitory inputs have the same degree of correlation *c*. Here we studied the effect of this parameter on the output firing rate. In our study, the spiking probability was higher for larger *c*, but too high values of *c* resulted in a “wasting” of input spikes and, therefore, reduced firing rates. Thus, as shown in Fig. 2a, an increase in *c* first leads to an increase and then to a decrease in the output firing rate, assuming a fixed total input strength (Bernander et al. 1994; Feng and Brown 2000; Kuhn et al. 2003; DeWeese and Zador 2006).

*In vivo* intracellular recordings have revealed strong correlation between excitatory and inhibitory inputs, while the temporal order of excitatory and inhibitory synaptic events could vary (Okun and Lampl 2008; Atallah and Scanziani 2009). Therefore, we considered in our model the effects of relative timing for fully coupled excitation and inhibition, that is, *c*_*EI*_=1. We found that when excitation preceded inhibition (*τ*_*ex*_<*τ*_*in*_) by 2 ms, the neuron could fire a spike before being inhibited. By contrast, when inhibition preceded excitation (*τ*_*ex*_>*τ*_*in*_) by 2 ms, the neuron was suppressed and became less likely to spike upon excitation, resulting in a lower output rate (Fig. 2b) (Sceniak and Sabo 2010; Zhou et al. 2010; Kremkow et al. 2010). Therefore, the relative timing between the excitatory and inhibitory synaptic events was crucial for the output rate of a neuron, especially for realistically small values of *c* (*c* ≤ 0.05 in our case).

Strong coupling between excitation and inhibition *c*_*EI*_ reduced the output activity when inhibition preceded excitation (Fig. 2d), but had only a small effect when excitation arrived first (Okun and Lampl 2008; Kremkow et al. 2010). This latter absence of an effect as shown in Fig. 2c is a consequence of inhibition arriving too late to suppress the output activity when it is preceded by correlated excitation.

The incoming compound PSPs revealed in the membrane potential recordings had different shapes and sizes (Okun and Lampl 2008). A temporally less sharp compound PSP reflects loose spike timing in the presynaptic pool of neurons (DeWeese and Zador 2006; Tiesinga et al. 2008). To mimic this, we jittered the spike times of all incoming inputs by a random value drawn from a zero-centered, uniform distribution of range *w* ms. When the excitation and inhibition were not coupled, the jitter only slightly reduced the output rate at small *c* (*c* ≤ 0.1 here), as shown in Fig. 2e. However, if the jitter was larger than the neuron’s refractory period, the neuron could spike more than once with strong enough excitation, leading to a second peak for higher values of *c*. When the two types of inputs were coupled, jittered inhibitory inputs could cancel jittered excitatory inputs more effectively, leading to strong reduction in firing rate (Fig. 2f). In general, the more jittered the input spikes were, the more the evoked PSPs spread over a longer time window, rendering the neuron less likely to spike, irrespective of the coupling strength of the two types of inputs.

### Correlation modulation by temporal structure of inputs

**Fig. 3:**
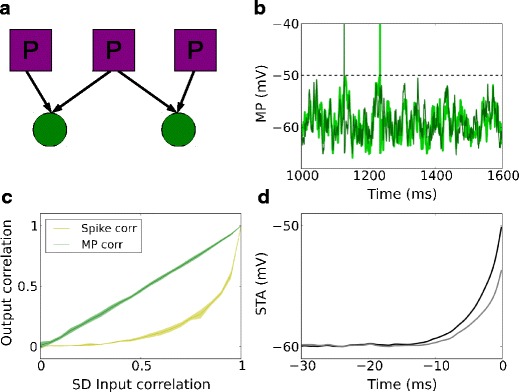
Output correlation of a neuron pair receiving correlated Poisson inputs. **a** Scheme of correlated Poisson inputs (both excitatory and inhibitory) to a pair of identical neurons. **b** Membrane potential traces of a pair of identical neurons receiving correlated Poisson inputs, with correlation coefficient 0.72. **c** Membrane potential correlation and output spike correlation as a function of input correlation. The membrane potential correlation is always higher than the output spike correlation, except at zero or full input correlation. **d** Spike-triggered average membrane potential for all spikes of one neuron in the pair (*black*) and the coincident membrane potential trace of the other neuron in the pair (*gray*), for an input correlation of strength 0.72

The output correlation of a pair of neurons receiving correlated inputs has been studied with regard to output spikes (Moreno-Bote and Parga 2006; de la Rocha et al. 2007; Shea-Brown et al. 2008; Tchumatchenko et al. 2010; Rosenbaum and Josic 2011) and membrane potentials (Kriener et al. 2008; Krumin and Shoham 2009). The typical theoretical treatment is to consider a pair of identical neurons receiving correlated Poisson inputs, as shown in Fig. 3a. The response is illustrated in Fig. 3b. The output spike correlation as measured by the Pearson correlation coefficient is found to be strictly lower than the input correlation, except for zero or full input correlation, where it is equal (Moreno-Bote and Parga 2006; de la Rocha et al. 2007; Shea-Brown et al. 2008), as shown in Fig. 3c. On the other hand, the membrane potential is simply the linear summation of its inputs and, thus, the membrane potential correlation is approximately equal to the input correlation.

High membrane potential correlation (Okun and Lampl 2008; Poulet and Petersen 2008; Gentet et al. 2010) but low output spike correlation (Gentet et al. 2010) have been observed in intracellular recordings from the somatosensory cortex *in vivo*, both in awake and in lightly anesthetized animals. This combination of properties can be accounted for by the typical two-neuron model using correlated Poisson input spike trains (Fig. 3c). However, recent experiments revealed that the spike-triggered average (STA) of membrane potentials preceding spike events recorded from neighboring excitatory neurons exhibits only a surprisingly small deflection (see Fig. 4e in Poulet and Petersen 2008 and Fig. 6G, left, in Gentet et al. 2010). This is inconsistent with the correlated Poisson input scenario: When a pair of neurons receives correlated Poisson input, the STA of the spiking neuron exhibits a rising phase just before the spike, whereas the membrane potential trace of the other neuron is also likely to display an upward deflection in the membrane potential, even though it may not necessarily spike itself. The deflection magnitude increases with increasing input correlation (data not shown). An illustration is given in Fig. 3d where the input correlation to a pair of excitatory neurons is 0.72 (Poulet and Petersen 2008) during quiet awake state. At such high input correlation, the deflection magnitude cannot be arbitrarily small in the shared Poisson input model. Observe that the upward deflection predicted by the theoretical treatment based on correlated Poisson processes is much stronger than that observed in experiments (Poulet and Petersen 2008; Gentet et al. 2010), indicating that the commonly used correlated Poisson model fails to describe this subtle feature in the physiological recordings. This experimental observation suggests that spikes are driven by strong, cell-specific (and, hence, uncorrelated) synaptic inputs, whereas the membrane potential fluctuations are caused by common or correlated inputs.

**Fig. 4:**
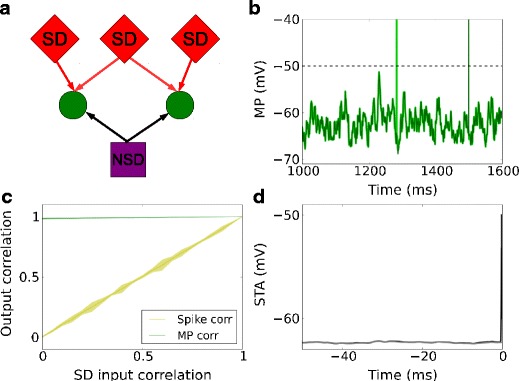
Output correlation of a neuron pair receiving common non-spike-driving (NSD) inputs. **a** Scheme of common NSD inputs (that is, NSD input correlation is 1) and correlated SD inputs to an identical neuron pair. **b** Membrane potential traces of the identical neuron pair receiving the inputs shown in (**a**). **c** Membrane potential correlation and output spike correlation as a function of correlation of SD inputs. **d** Spike-triggered average membrane potential for all spikes of one neuron in the pair (*black*) and the coincident membrane potential trace of the other neuron in the pair (*gray*), for SD input correlation 0 and NSD input correlation 1

Therefore, we argue for the separation of spike-driving (SD) and non-spike-driving (NSD) inputs to account for the above phenomenon. The SD inputs represent coincident synaptic events (or, alternatively, strong synapses) that can effectively evoke a spike. These two inputs are modeled here by a MIP process with big enough within-pool correlation *c* to evoke a spike reliably, and a low-intensity Poisson process which mainly contributes to the membrane potential fluctuations, respectively. A combination of shared NSD inputs and uncorrelated SD inputs together (Fig. 4a) gives rise to correlated subthreshold membrane potential and uncorrelated output spiking. An example with common NSD inputs is shown in Fig. 4b. Figure 4c displays the output spike and membrane potential correlations, as a function of SD input correlation. Observe that the output spike correlation increased linearly with SD input correlation, whereas the membrane potential correlation stayed almost the same as the NSD input correlation, which is 1. In addition, with such an input configuration, STA membrane potentials preceding spikes (Fig. 4d) in neighboring excitatory neurons showed a very small deflection.

**Fig. 5:**
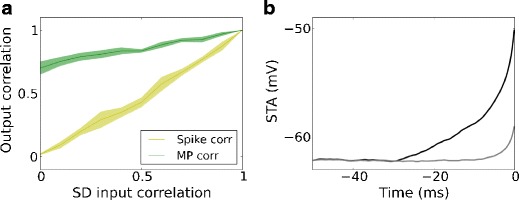
Output correlation of a neuron pair receiving common non-spike-driving (NSD) inputs. Similar to Fig. 4, except that the temporal displacements of input spikes are drawn from a uniform distribution of width 30 ms to resemble excitatory neurons in the barrel cortex. **a** Membrane potential correlation and output spike correlation as a function of correlation of SD inputs. The membrane potential correlation generally decreases for SD input correlation compared to Fig. 4c because of the jitter. The membrane potential correlation is 0.72 at SD input correlation 0. **b** Spike-triggered average membrane potential for all spikes of one neuron in the pair (*black*), and the coincident membrane potential trace of the other neuron in the pair (*gray*), for SD input correlation 0 and NSD input correlation 1

The results in Fig. 4 are based on precisely timed input spikes. Figure 5a shows the corresponding output spike correlations and membrane potential correlations for uniformly jittered input spikes of both NSD and SD inputs, using a jitter window size of 30 ms. The output spike correlation scaled linearly with the SD input correlation, whereas the membrane potential correlation was generally smaller compared to Fig. 4c, due to the jitter. At zero SD input correlation, a membrane potential correlation of 0.72 could be achieved, which is approximately the value observed during the state of quiet wakefulness in mice (Poulet and Petersen 2008). Furthermore, the membrane potential preceding a spike in the model neuron, shown in Fig. 5b, was consistent with the experimental findings: the membrane potentials preceding a spike in neighboring excitatory neurons in the barrel cortex (Poulet and Petersen 2008; Gentet et al. 2010) exhibited a smaller deflection of the STA than the standard Poisson model with otherwise identical parameters (Fig. 3c). Jittering the coincident input spikes contributed to the properties of the subthreshold membrane potential preceding a spike, as well as to the increased uncertainty in output spike timing. The superposition of common NSD and uncorrelated SD inputs between the two neurons gave rise to the small deflection. The amplitude of the STA at the spike trigger (time 0) is at the threshold of the model neuron. Prior to the trigger, the STA without jitter displays the average membrane potential due to background inputs, whereas the STA with jitter is additionally affected by the contribution of the jittered spikes from SD inputs. The total amount of input is the same in both cases. We obtained similar results as those described above at a membrane potential correlation of 0.33, which is approximately what is observed during the whisking state in experiments (Poulet and Petersen 2008), except that the upward deflection of the STA was smaller (data not shown).

**Fig. 6:**
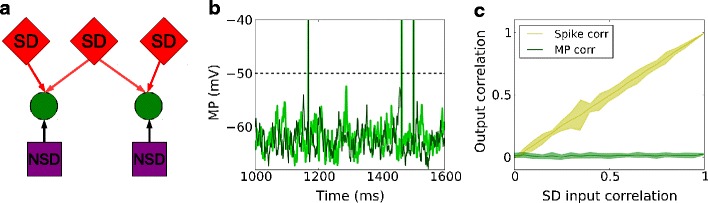
Output correlation of a neuron pair receiving independent NSD inputs. **a** Scheme of independent NSD inputs and correlated SD inputs to an identical neuron pair. **b** Membrane potential traces of the identical neuron pair receiving the inputs shown in (**a**). **c** Membrane potential correlation and output spike correlation as a function of correlation of SD inputs

Figure 6a shows the scheme of an input configuration commonly adopted in computational models, in which the same SD input arrives at multiple neurons, while individual neurons receive independent NSD inputs. In this case, the membrane potential is weakly correlated, but the information carried by the spikes is transmitted reliably (Fig. 6b). One typical example of this scenario is the feedforward network operating in the synfire mode (Diesmann et al. 1999; Kumar et al. 2010). While the embedding of feedforward networks within a recurrent network can render the background activity to be synchronous (Kumar et al. 2008), their pairwise correlations are captured by Fig. 4c when the output spike correlation approaches unity.

Spike and membrane potential correlations can be tuned separately, provided the inputs comprise both SD and NSD inputs. Such segregation of SD and NSD inputs may well explain the correlations of excitatory neurons in the barrel column, as presented here. SD and NSD inputs mainly contribute to spike and membrane potential correlations, respectively, if SD inputs are likely to evoke a spike while NSD inputs are not. Figures 4c and 6c showed that the membrane potential correlation depends mainly on the NSD input correlation, but is insensitive to SD input correlation. By contrast, spike correlation increases linearly with SD input correlation, irrespective of whether NSD input correlation is small (close to 0) or large (close to 1).

## Discussion

Response rate and correlation are fundamental descriptors of neuronal activity widely investigated in neuronal dynamics. Here, we explored the effect of input correlations on the output firing rate of single neurons, and on the output correlation of pairs of neurons, both at the level of membrane potentials and of output spike trains.

### Factors that potentially modulate firing rates

Based on the *in vivo* intracellular recordings in the somatosensory cortex, we set up a minimal neuron model and an input configuration that can capture key features of subthreshold and suprathreshold neuronal dynamics (Okun and Lampl 2008). Our simple point neuron model employs its excitatory and inhibitory inputs upon current injection in a way similar to what has been shown in experiments (Fig. 1c). We further examined several rate modulating factors deduced from experimental findings: the strength of E-I coupling (Salinas and Sejnowski 2000; Wilent and Contreras 2005; Okun and Lampl 2008), the relative timing between the E-I (Okun and Lampl 2008; Atallah and Scanziani 2009; Cafaro and Rieke 2010), and the degree of jitter of input spikes (Svirskis and Rinzel 2000; Moreno et al. 2002; Okun and Lampl 2008). E-I correlation can change in different cognitive states (Okun and Lampl 2008; Gentet et al. 2010). The lag between excitation and inhibition shows a range of values from positive to negative (Okun and Lampl 2008; Atallah and Scanziani 2009; Cafaro and Rieke 2010). Moreover, the shapes and sizes of individual compound EPSPs and IPSPs differ, indicating that input spikes are subject to temporal jitter. With computational modeling based on numerical simulations, we found that the interplay of multiple potential rate modulating factors as illustrated in our study can lead to marked changes in the firing rate of the receiving neuron. Yet, any single factor, e.g. the relative timing of E-I inputs or the coincidence of E-E or I-I inputs alone may be ineffective to alter the spiking activity of a neuron.

The LIF neuron model serves as a minimal model for many theoretical studies. Although this model cannot capture all the details of a neuron such as dendrite topology and active conductance dynamics, our implementation of input structure provides extended interpretations to the usual LIF neuron model in response to correlated inputs. For instance, when synapses are distributed over long dendritic branches, synaptic inputs due to coincident spikes appear as jittered inputs at the soma. The extent of jitter depends on how widely the synapses are distributed, on dendritic morphology and on the distribution of voltage-dependent ion channels. Another example is that a neuron with differential localization of excitatory and inhibitory synapses may integrate excitatory and inhibitory inputs in different temporal order at the soma, compared to a neuron with identical (or largely overlapping) distributions of both synapse types, in response to coincident inputs. The relative timing between the E-I inputs can account for this effect.

### Inference of input structure from spike and membrane potential correlations

It has been shown with *in vivo* recordings in the rat barrel cortex that the membrane potentials of excitatory and inhibitory neurons are highly correlated (Poulet and Petersen 2008; Gentet et al. 2010). The membrane potential distribution in the auditory cortex is largely non-Gaussian and inputs arrive in the form of “activity bumps” (DeWeese and Zador 2006). These findings imply that neurons are confronted with correlated inputs, which can be due to common sources of inputs, or to coincident inputs from different sources. By contrast, the output spike correlation between excitatory neurons is quite small compared to the membrane potential correlation. Earlier studies have addressed this scenario by providing correlated inputs to pairs of neurons (Fig. 3a) (Moreno-Bote and Parga 2006; de la Rocha et al. 2007; Shea-Brown et al. 2008; Krumin and Shoham 2009; Tchumatchenko et al. 2010). The output spike correlation is then a specific function of the input spike correlation, as shown in Fig. 3c. These results may apply to the correlation between inhibitory neurons, as well as between excitatory and inhibitory neurons, but not between excitatory neurons. The reason is that in experimental recordings the STA traces of membrane potentials preceding spike events for neighboring excitatory neurons show a very small deflection (Poulet and Petersen 2008; Gentet et al. 2010), in contrast to the prediction from a shared Poisson model, as shown in Fig. 3d.

Here we introduced the notion of spike driving (SD), and non-spike driving (NSD) inputs. The latter only contribute to the subthreshold membrane potential at low firing rates (around 1 Hz) and in the absence of spike jitter, whereas the SD input correlation transfers to the output spike correlation. In the presence of spike jitter, we expect that these relations are slightly off, for example, Fig. 5a shows that the output membrane potential correlation drops to around 0.72 at full NSD input correlation for a spike jitter window size of 30 ms.

We found that output spike correlation and membrane potential correlation can be manipulated separately by SD and NSD inputs. In response to uncorrelated SD inputs and correlated NSD inputs, a neuron pair may display correlated membrane potentials, but uncorrelated action potentials. In addition, when one neuron exhibits a strong depolarization leading to a spike, the membrane trace of another neuron is still independent of such depolarization (Fig. 4d). A stronger deflection is expected when the input leading to a spike is correlated with the input to the non-spiking neuron. Our suggestion that the SD inputs to individual excitatory neurons are (nearly) independent fits the observations made in recordings of nearby excitatory neurons in the barrel cortex, and is consistent with the proposal that such spikes are driven by large, rapid and cell-specific synaptic inputs (Gentet et al. 2010).

Spike and membrane potential correlation can shed light on the temporal structure of the inputs to pairs of neurons. Regarding layer 2/3 of the barrel cortex, all neurons are likely to receive relatively weak synaptic inputs from the local network, which can be modeled as correlated NSD inputs, resulting in correlated membrane potentials. Inhibitory neurons are more excitable than excitatory neurons, so they may spike more often in response to the local inputs, also reflected in their spike correlation. By contrast, excitatory neurons are driven to spike by infrequent, but strong, cell-specific synaptic inputs, which can be modeled as uncorrelated SD inputs. Then the next question is: where do such SD inputs come from?

In fact, L2/3 of the barrel cortex receives feedforward inputs from L4, the thalamus, motor cortex M1 and other higher order cortical regions (Petreanu et al. 2009). It is conceivable that the projections could be topographically mapped such that individual L2/3 neurons receive distinct feedforward inputs. Given that the cortical connectivity is sparse (Braitenberg and Schüz 1991; Binzegger et al. 2004) and the spike correlation is low (Gentet et al. 2010; Ecker et al. 2010; Renart et al. 2010), it would seem that cortical neurons are not likely to receive strongly correlated inputs. However, in view of the massive number of neurons in the cortex, a connection probability and/or spike correlation in the range of 0.01 may nevertheless result in a significant amount of coincident spikes (Binzegger et al. 2004; Boucsein et al. 2011). Thus, low spike correlation may be compensated by a huge pool of presynaptic neurons.

Moreover, correlated inputs in our model can also be interpreted as strong synapses (Song et al. 2005) or as highly synchronized clusters of input spikes (Léger et al. 2005), as in the synfire mode in feedforward networks (Kumar et al. 2010). That is, a few strong synapses or, alternatively, a highly synchronous spike volley can make a receiving neuron spike reliably. In fact, thalamic input that drives spiking in the barrel cortex is likely to arise from weak but numerous correlated inputs (Bruno and Sakmann 2006). Hence, such strong synapses and inputs similar to synfire volleys can both act as the SD inputs, whereas all weak synapses and asynchronous inputs are more likely to constitute the NSD inputs. In such a scenario, our model suggests that strong synapses (or, alternatively, synfire volleys) in the rodent barrel cortex arise from independent sources. Therefore, as we have demonstrated, spike and membrane potential correlations can provide crucial hints about the organization of the local network when interpreted according to our scheme of SD and NSD inputs.

Recently, Schultze-Kraft et al. (2013) examined the correlation transfer when a neuron pair receives independent Poisson inputs and shared MIP input. By contrast, we studied two scenarios: in Fig. 4, the neuron pair received common NSD (Poisson) inputs, with the SD (MIP) input correlation varying between 0 and 1; in Fig. 6, the neuron pair received independent NSD (Poisson) inputs, with the SD (MIP) input correlation varying between 0 and 1. In particular, the setting when NSD (Poisson) input is common but the SD (MIP) inputs are independent, as shown in Fig. 5, can explain the STA membrane potentials of excitatory neuron pairs observed in experiments (Poulet and Petersen 2008; Gentet et al. 2010).

### Implications of distinguishing between SD and NSD inputs

When the input (which could be coincident spikes on multiple synapses or, alternatively, single input spikes on very strong synapses) triggers an output spike with high probability, we refer to it as a SD input. Otherwise, the input is called NSD. Such classification can explain the output correlation of excitatory neurons in the barrel cortex (Poulet and Petersen 2008; Gentet et al. 2010). In this context, inhibitory inputs are clearly NSD. Excitatory inputs which do not lead to spiking, including those preceded by inhibitory inputs, preventing the neurons from responding to excitation, are considered NSD inputs. Correlated excitatory inputs are natural candidates for being SD input. However, we have shown in Fig. 2 that the interaction between correlated excitation and inhibition can render even correlated excitatory inputs insufficient to elicit spikes in the postsynaptic neuron. Thus, a change in the balance or the relative timing between excitatory and inhibitory inputs can dynamically change inputs from SD to NSD type. Interestingly, knowing the correlations between membrane potentials and spikes can help us delineate the nature of SD and NSD.

**Fig. 7:**
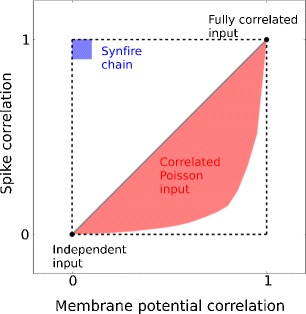
Summary of spike and membrane potential correlations for different input scenarios. The area bounded by the dashed lines represents the correlation regimes accessible by the separation of SD and NSD inputs. Further explanation in the main text (cf. Section 4)

In addition to the possibility of explaining the difference in STA profiles prior to spiking in neighboring excitatory neurons in L2/3 rodent barrel cortex, the separation between SD and NSD inputs creates further possible scenarios regarding spike and membrane potential correlations, schematically summarized in Fig. 7. The correlated Poisson input model can only provide a limited range of these two correlations, shaded in red. Here, spike correlation is always smaller than or equal to membrane potential correlation, with equality only holding for “parrot neurons” which transmit every input spike into an output spike (portrayed as the gray line along the diagonal). By contrast, the area bounded by the dashed lines represents the regime of combinations of spike and membrane correlations accessible by the separation of SD and NSD inputs. Note that its spike-generating mechanism is different from the correlated Poisson model, therefore the correlated Poisson model is not a special case. In particular, the scenario in which SD inputs are correlated but NSD is not, is similar to the propagation of spiking activity in feedforward networks operating in the so-called “synfire mode” (Kumar et al. 2008, 2010), shaded in blue in Fig. 7.

In the context of our study, we specifically distinguished between SD and NSD inputs. As can be concluded from Figs. 4c and 6c, the correlation of SD inputs determines the output spike correlation, whereas the correlation of subthreshold inputs determines the membrane potential correlation. In reality, the distinction between SD and NSD inputs may not be so clear-cut. For example, coincident inputs may cause one neuron to spike, but not the other one, and a neuron can spike after temporal integration of sufficient NSD inputs. Nonetheless, our approach provides new important insights into the relation between spike correlations and membrane potential correlations in experimental and computational studies. To conclude, our work establishes insightful relations between the temporal structure of neuronal input and output activity.
